# The Impact of Human Milk Feeding on Long-Term Risk of Obesity and Cardiovascular Disease

**DOI:** 10.1089/bfm.2019.0037

**Published:** 2019-04-12

**Authors:** Atul Singhal

**Affiliations:** ^1^ Institute of Child Health, University College London, London, United Kingdom.; ^2^ Great Ormond Street Hospital, London, United Kingdom.

Beyond just meeting nutritional requirements, nutrition during infancy is linked to disease later in life, a concept known as “the developmental origins of health and disease.” As early as the 1930s, study in rodents demonstrated that early calorie restriction increased the life span by 35% and reduced the incidence of tumors, kidney disease, vascular calcification, and chronic pneumonia.^1^ In the 1960s, studies in rats showed that early nutrition during the first 3 weeks had a life-long impact on body size; rats that received less milk during this time continued to weigh less throughout life compared with rats that received more milk.^2^ Further evidence for nutritional programming came from studies in baboons, which showed that overfeeding during infancy increased the tendency for obesity in adulthood.^3^

One of the first studies to demonstrate nutritional programming in humans showed that rapid weight gain from as early as the first 6 weeks of life was associated with obesity at 8 years of age.^4^ Beginning in the late 1980s, observational studies linking low birth weight with later cardiovascular risk factors and diabetes shifted focus to nutrition during the fetal period, resulting in the “fetal origins of adult disease” hypothesis.^5^ Subsequent studies have suggested that, although a high-quality maternal diet can prevent infants born small for gestational age (SGA) and low birth weight, dietary supplementation during pregnancy may not affect the offspring's risk of obesity and cardiovascular disease.^6–9^ Other studies suggest prepregnancy obesity and maternal weight gain are associated with obesity in the offspring; however, there has been no convincing evidence of a causal effect of these factors on offspring obesity.^10–12^

There have been several systematic reviews supporting the positive effects of breastfeeding on cardiovascular risk factors, such as obesity and type 2 diabetes.^13,14^ Experimental trials in preterm infants assigned to donor human milk or formula demonstrated the beneficial effects of human milk on blood pressure, cholesterol, obesity, and insulin resistance.^15–18^ One possible explanation for the benefits of human milk on cardiovascular risk may be the slower growth of infants receiving breast milk versus those receiving formula (i.e., the growth acceleration hypothesis).^19^ Human milk has lower energy and protein content than formula, resulting in slower weight gain during infancy with human milk versus formula. Faster infant growth has been associated with later obesity in many studies, including five randomized trials.^19^ A randomized trial of high nutrient intake in term SGA infants showed that infants fed enriched formula had significantly higher diastolic blood pressure and fat mass at 6–8 years compared with those fed standard formula.^20,21^ Similarly, a multicenter randomized trial by the European Childhood Obesity Group reported a significantly higher body mass index and a 2.4 times greater risk of becoming obese at 6 years of age in infants who had been fed a high-protein formula versus those fed a low-protein formula in infancy.^22^ It has been hypothesized that accelerated growth in infancy may result in hormonal changes that program a higher set point for appetite, leading to higher food intake throughout life.^23^ Bottle-fed infants may be less likely to regulate milk intake or appetite later in infancy; infants receiving human milk from a bottle were 67% less likely to have a high satiety response.^24^

In summary, there is strong evidence supporting the benefits of breastfeeding and human milk consumption during infancy for long-term cardiovascular health. These benefits may be related to the slower early growth of infants receiving human milk or lower-protein formulas versus those fed standard formulas. Preventative strategies, such as promoting breastfeeding, reducing the protein content of formula, avoiding overnutrition, and encouraging responsive bottle feeding (i.e., recognizing feeding cues), may help to slow the rate of weight gain in infancy and reduce the long-term burden of cardiovascular disease.
